# Association of chronic diseases and lifestyle factors with suicidal ideation among adults aged 18–69 years in Eswatini: evidence from a population-based survey

**DOI:** 10.1186/s12889-021-12302-6

**Published:** 2021-12-10

**Authors:** Mfundi President Sebenele Motsa, Hung-Yi Chiou, Yi-Hua Chen

**Affiliations:** 1 PhD Program in Global Health and Health Security, College of Public Health, Taipei Medical University, 250 Wuxing St., Taipei City, 110 Taiwan; 2 School of Public Health, College of Public Health, Taipei Medical University, 250 Wuxing St., Taipei City, 110 Taiwan; 3 Institute of Population Health Sciences, National Health Research Institutes, Zhunan Town, Miaoli County 35053 Taiwan

**Keywords:** Suicidal ideation, Chronic disease, Lifestyle, Exercise

## Abstract

**Background:**

How chronic diseases and lifestyle affect suicidal ideation in the sub-Saharan region remains unclear. We investigated the association of chronic diseases and lifestyle with suicidal ideation in the past year and the potential modifying role of sociodemographic status on this association. The findings can guide suicide prevention interventions.

**Methods:**

We analyzed 3026 respondents from the World Health Organization STEPwise approach to noncommunicable disease risk factor surveillance conducted in Eswatini in 2014. The outcome was past-year suicidal ideation, and the main predictors were chronic diseases and lifestyle. Multiple logistic regression was used to estimate predictors, and subgroup analysis was performed to assess effect modification.

**Results:**

The prevalence of past-year suicidal ideation was 9.9%. After adjustment for covariates, including sex, marital status, employment status, and education level, individuals aged 18–30 years (adjusted odds ratio [aOR]: 2.27, 95% confidence interval [CI]: 1.22–4.22) were more likely to have had past-year suicidal ideation than those aged 45–69 years. After adjustment for covariates among employed individuals, having high blood pressure (aOR: 3.38, 95% CI: 1.54–7.40), not exercising (aOR: 2.65, 95% CI: 1.09–6.39), drinking alcohol (aOR: 2.40, 95% CI: 1.14–5.05), being aged 18–30 years (aOR: 3.50, 95% CI: 1.01–12.1), and being exposed to threats (aOR: 2.37, 95% CI: 1.01–5.53) were significantly associated with past-year suicidal ideation.

**Conclusions:**

Among currently employed individuals, having high blood pressure, not exercising, and drinking alcohol were associated with past-year suicidal ideation. The findings highlight the importance of developing and strengthening systems for early identification of suicidal ideation risk.

## Introduction

Suicide is a major global health concern and a substantial contributor to mortality worldwide; yet, it is preventable [1]. One person is estimated to commit suicide every 40 s, and approximately 79% of these deaths occur in developing countries [2]. In general, not all people with suicidal ideation will attempt suicide, but on the continuum of suicidal behavior, it is a first step [3]. The number of individuals who engage in suicidal behaviors is worrisome [2], and this problem could be addressed through early identification of suicidal ideation [4]. Suicidal ideation entails thinking about, considering, and planning suicide [4–6]. Moreover, suicidal ideation is the first step on the suicidal behavior continuum, and knowledge regarding the transition from ideation to action helps form prevention strategies [7].

The estimated global lifetime prevalence of suicidal ideation is 9.2% [7], and for low- and middle-income countries (LMICs), the lifetime prevalence of suicidal ideation is higher (10.3%) [8]. Although the World Health Organization (WHO) has recommended a mental health gap action program to reduce suicides in member states [9], the magnitude of the problem remains high; estimates indicate that suicides account for 1.42% (1.33–1.46%) of overall deaths per 100,000 people globally, with the highest proportion in Europe (1.53%; 1.48–1.58%); in Africa, it is 0.87% (0.79–0.96%) [1]. Most cross-national studies on suicidal ideation focused on the adult population have investigated participants from developed countries [6, 10]; few have involved the sub-Saharan region (SSA) [11].

Many factors are associated with suicidal ideation, and some variations have been observed between regions and countries with respect to sociodemographic variables, comorbidities, and lifestyle factors [12–14]. A meta-analysis of studies from European countries suggested that the number of chronic health conditions is associated with suicidal ideation among adults [10]. Additionally, evidence from developed countries has suggested that physical health problems, including high blood pressure (HBP) [15–17] and cardiovascular disease (CVD) [18], are positively associated with suicidal ideation. Similarly, literature from developing countries has revealed that physical illness [11, 19, 20] and CVD [20] are associated with suicidal ideation.

Studies from developing countries have reported an association between lifestyle factors (smoking, drinking alcohol, and exercise) and suicidal ideation among adults [21, 22], as have studies from developed countries [23]. A meta-analysis indicated that physical exercise is negatively associated with suicidal ideation [24]. A South African case–control study reported that alcohol increases the chances of hospitalization from suicidal behavior [25]; however, this study focused on suicide attempts and not ideation.

Suicide is among the leading causes of mortality in Eswatini, with an estimated age-standardized rate of 16.7 per 100,000 persons in 2016 [26]. A study conducted in Eswatini among school-going adolescents revealed that age, sex, food insecurity, and sexual behavior were associated with suicidal behavior [27]. However, population-based factors that influence the adult population are unclear. Another study from Eswatini involving 15–69-year-old adults indicated that childhood and adult sexual abuse and threats were associated with suicidal ideation [28]. However, this study had the following limitations: it included adolescents in the study sample, which may have inflated the suicidal ideation risk because if ideation begins during adolescence, it may persist throughout adulthood if left unaddressed [29]; it defined the outcome as a combination of suicidal thoughts, plans, and recent suicide attempts, which is nonspecific; and it focused on abuse-related factors rather than the effects of chronic diseases and lifestyle factors.

Although some studies have addressed the influence of chronic diseases and lifestyle factors on suicidal ideation, few have focused on Eswatini. The mortality rates attributable to noncommunicable diseases in Eswatini are high, estimated at 37% in 2016 [30]. Therefore, investigating the association of chronic diseases and lifestyle with suicidal ideation is crucial for improving intervention strategies against suicidal ideation. In addition, examining how socioeconomic status (SES) is a potential effect modifier on suicidal ideation is crucial to identify high-risk groups. Studies have considered suicidal behavior in general, but few have focused on past-year suicidal ideation. To address this gap in the literature, we investigated, first, the association of chronic diseases and lifestyle with past-year suicidal ideation and, second, the potential modifying role of SES in this association.

## Methods

### Study design and data source

This study involved the secondary analysis of data obtained from the WHO STEPwise approach to surveillance (STEPS) conducted in Eswatini in 2014 [31], which was designed to collect data on noncommunicable disease risk factors at the population level among adults aged 15–69 years. The selection procedure for the survey is detailed elsewhere [32]. Briefly, the STEPS employed a multistage cluster sampling design. In total, 216 primary sampling units (PSUs) were selected using probability-proportionate-to-size sampling, and approximately 20 households were systematically sampled from the PSUs to form secondary sampling units. A Kish sampling method was used to identify an eligible person from each household. Data were collected for 32 days from November to December 2014. Participants were adult men and women aged 15–69 years from all four regions of Eswatini.

The overall sample size of STEPS was 3534; however, for this analysis, we excluded 275 individuals aged < 18 years and 233 individuals who did not answer suicidal ideation questions. Our final sample had 3026 participants aged 18–69 years who responded to questions on suicide, with the answers used to ascertain whether they had suicidal thoughts. We focused on this age group because studies have examined factors associated with suicidal behavior among adolescents and adults but have not evaluated adult-only factors, which may be different [27, 28]. Furthermore, we examined differences in sociodemographics (SES) and the key factors of chronic diseases (HBP and CVD) and lifestyle (lifetime alcohol use, currently smoking, and exercise) between those who responded (n = 3026) and those who did not respond (n = 233) to questions on past-year suicidal ideation. No significant differences were noted between the two groups.

### Outcome variable

The outcome of interest in this analysis was past-year suicidal ideation. In this study, past-year suicidal ideation was defined as a respondent’s feelings or thoughts regarding killing themselves. It was determined based on the following item in STEPS: “During the past 12 months, have you seriously considered attempting suicide?” The response options were “yes” and “no,” and we recorded past-year suicidal ideation yes and suicidal ideation no as 1 or 0, respectively.

### Independent variables

The main independent variables in this study were chronic diseases (i.e., HBP and CVD) and lifestyle factors (i.e., exercise, lifetime alcohol use, and currently smoking). HBP was assessed using the item “Have you ever been told by a doctor or other health worker that you have raised blood pressure or hypertension?” CVD was assessed using the item “Have you ever had a heart attack or chest pain from heart disease (angina) or stroke (cerebrovascular accident or incident)?” Exercise was assessed using the item “Do you do any moderate-intensity sports, fitness activities, or recreational (leisure) activities that cause a small increase in breathing or heart rate such as brisk walking, cycling, swimming, and volleyball for at least 10 min continually?” Lifetime alcohol use was assessed using the item “Have you ever consumed any alcohol such as beer, wine, spirits, or other local variants?” Currently smoking was assessed using the item “Do you currently smoke any tobacco products, such as cigarettes, cigars, and pipes?”

### Covariates

Abuse-related factors were considered covariates in this study because of their effects on our main variables [33]. Childhood sexual abuse was assessed by asking, “Looking back on your childhood, did an adult or anyone at least 5 years older than you ever touch you sexually or try to make you touch them sexually or force you to have sex?” Adult sexual abuse was assessed by asking, “Since your 18th birthday, have you ever experienced a sex act involving vaginal, oral, or anal penetration against your will?” Threat was determined by asking, “In the past 12 months, have you been frightened for your safety or that of your family because of the anger or threats of another person(s)?” Family history of a suicide attempt was assessed by asking, “Has anyone in your close family (parent, sibling, or children) ever attempted suicide?”

### Potential effect modifiers

Using the literature [6, 34, 35] and information of the Eswatini STEPS [32], we examined sociodemographic factors as potential effect modifiers. The variables were age (18–30, 31–45, and 46–69 years), sex (male and female), marital status (married, never married, and separated/divorced), educational level (primary and lower, secondary or lower, high school and higher), employment status (currently employed and currently unemployed), and residence (rural and urban).

### Statistical analysis

Stata version (2017): Release 15 (StataCorp LP, College Station, TX, USA) was used for the analyses. We applied weights by using the *svy* command to account for the complex STEPS design and for clustering, stratification, and nonresponses. We assessed the prevalence of past-year suicidal ideation for the entire study population. Variables are presented in terms of their frequency and percentage. Furthermore, we assessed bivariate associations between independent variables and past-year suicidal ideation by using the chi-square test. We assessed the unadjusted and adjusted associations of chronic diseases and lifestyle factors with past-year suicidal ideation by using logistic regression to estimate the odds of having past-year suicidal ideation. Our results are presented in terms of crude odds ratios and adjusted odds ratios (aORs) along with their 95% confidence intervals (CIs). Statistical significance was indicated if *p* < 0.05.

Interactions of chronic diseases and lifestyle factors with sociodemographic factors on past-year suicidal ideation were examined, and the interaction terms were considered significant if *p* < 0.1 [36], in which case a subgroup analysis (stratification) was used for assessment. Sociodemographic characteristics were examined to assess potential modifying effects and were included in the model as a covariate for adjustment.

### Ethical considerations

The survey complied with the standards governing the protection of human participants, and written informed consent was obtained from each respondent. The Swaziland Ethics Committee approved the initial STEPS, and the custodian of the data was notified about the intention to analyze the survey. Permission to analyze data was obtained from the WHO Non-communicable diseases (NCD) Microdata Repository, which serves as the custodian of publicly available data [31].

## Results

### Distribution of participants by factors associated with past-year suicidal ideation

The data of 3026 participants were analyzed. Table 1 presents their characteristics and the distribution of past-year suicidal ideation according to independent variables. The overall prevalence of past-year suicidal ideation was 9.9%. Chronic disease burden was high, with HBP and CVD at 25.7% and 13.1%, respectively. Individuals with past-year suicidal ideation tended to have a history of CVD, have a history of lifetime alcohol use, be women, be currently unemployed, have a family history of suicide, and have experienced childhood sexual abuse, adult sexual abuse, or threats (all *p* < 0.05).

**Table 1:** Chronic diseases and lifestyle factors associated with suicidal ideation

Characteristics	Total (N = 3026)	Suicidal Ideation		*p* value
	**n (%)**	**Yes** **n (%, weighted)**	**No** **n (%, weighted)**	
**Chronic disease**				
** High blood pressure**				0.45
Yes	582 (25.7)	63 (28.7)	519 (25.4)	
No	1363 (74.3)	133 (71.3)	1230 (74.6)	
** Cardiovascular disease**				0.04*
Yes	102 (13.1)	26 (7.7)	105 (4.6)	
No	644 (86.9)	272 (92.3)	2623 (95.4)	
** Lifestyle factors**				
** Exercise**				0.13
Yes	516 (21)	44 (16.6)	472 (21.5)	
No	2510 (79)	254 (83.4)	2256 (78.5)	
** Currently smoking**				0.13
Yes	191 (6.9)	25 (9.9)	166 (6.6)	
No	2835 (93.1)	273 (90.1)	2562 (93.4)	
** Lifetime alcohol use**				0.04*
Yes	789 (28.8)	98 (35.3)	691 (28.0)	
No	2237 (71.2)	200 (64.7)	2037 (72.0)	
**Demographics**				
** Age (years)**				0.25
18–30	1182 (52.2)	124 (56.7)	1058 (51.7)	
31–45	857 (26.4)	91 (25.9)	766 (26.4)	
46–69	987 (21.4)	83 (17.4)	904 (21.9)	
** Sex**				<0.001*
Women	2010 (55.7)	227 (68.7)	1783 (54.3)	
Men	1016 (44.3)	71 (31.3)	945 (45.7)	
** Marital status**				0.17
Married	1236 (51.8)	110 (49.4)	1126 (52.1)	
Never married	1383 (38.6)	135 (36.9)	1248 (38.8)	
Separated/divorce	405 (9.5)	52 (13.7)	353 (9.1)	
** Education level**				0.94
Primary and lower	971 (24.6)	100 (23.8)	871 (24.6)	
Secondary	1260 (44)	136 (45.2)	1124 (43.9)	
High school and higher	792 (31.4)	61 (31)	731 (31.5)	
** Employment status**				0.02*
Currently employed	1229 (39.7)	99 (31.7)	1130 (40.6)	
Currently unemployed	1715 (60.3)	190 (68.3)	1525 (59.4)	
** Residence**				0.90
Rural	2205 (71.3)	218 (71)	1987 (71.4)	
Urban	821 (28.7)	80 (29)	741 (28.6)	
** Family history of suicide**				0.002**
Yes	320 (11.9)	48 (19.8)	272 (11.0)	
No	2704 (88.1)	248 (80.2)	2456 (89.0)	
**Abuse factors**				<0.001**
** Childhood sexual abuse**				
Yes	175,519 (5)	40 (14.4)	135 (4)	
No	2840 (95)	258 (85.6)	2582 (96)	
** Adult sexual abuse**				<0.001**
Yes	113 (3.3)	24 (9.3)	89 (2.6)	
No	2898 (96.7)	274 (90.7)	2624 (97.4)	
** Threats**				<0.001**
Yes	388 (13.9)	75 (25.5)	313 (12.6)	
No	2635 (86.1)	223 (74.5)	2412 (87.4)	

* *p* value from chi square and Fishers exact tests *p < 0.05; **p < 0.01; ***p < 0.005

### Factors associated with past-year suicidal ideation

 Table 2 presents the results of univariate, multivariable logistic, and stratified models that were used to examine factors associated with past-year suicidal ideation. After adjustment for sex, marital status, residence, current employment status, education level, HBP, CVD, currently smoking, lifetime alcohol use, childhood sexual abuse, adult sexual abuse, and threats, individuals aged 18–30 years were more likely to have past-year suicidal ideation (aOR = 2.27, 95% CI = 1.22–4.22) than those aged 46–69 years. Individuals with a family history of suicide were also more likely to have past-year suicidal ideation (aOR = 1.92, 95% CI = 1.24–3.09) than those without. In addition, individuals who experienced childhood sexual abuse or threats were more likely to have past-year suicidal ideation (aOR = 2.26, 95% CI = 1.10–4.66 and aOR = 1.88, 95% CI = 1.15–3.06, respectively) than those who did not. Chronic diseases and lifestyle factors were not associated with past-year suicidal ideation in the overall group.

**Table 2:** Association of chronic diseases and lifestyle factors with suicidal ideation, stratified by age

Characteristics	Total		Age 18–30 years	Age 31–45 years	Age 46–69 years
	cOR (95% CI)	aOR^a^ (95% CI)	aOR^b^ (95% CI)	aOR^b^ (95% CI)	aOR^b^ (95% CI)
**Chronic disease**					
** High blood pressure**					
Yes	1.18 (0.76–1.83)	1.62 (0.97–2.72)	1.08 (0.48–2.42)	3.28 (1.36–7.88) **	1.22 (0.54–2.75)
No	1	1	1	1	1
** Cardiovascular disease**					
Yes	1.74 (1.01–2.98) *	1.45 (0.74–3.82)	1.22 (0.40–4.39)	2.68 (0.58–12.2)	0.68 (0.23–1.97)
No	1	1	1	1	1
**Lifestyle factors**					
** Exercise**					
No	1.35 (0.89–2.08)	1.51 (0.83–2.73)	1.58 (0.70–3.58)	0.94 (0.34–2.60)	1.24 (0.34–4.45)
Yes	1	1	1	1	1
** Currently smoking**					
Yes	1.56 (0.86–2.82)	1.39 (0.58–3.36)	0.92 (0.25–3.32)	0.92 (0.09–8.79)	1.75 (0.39–7.77)
No	1	1	1	1	1
** Lifetime alcohol use**					
Yes	1.40 (1.00–1.94) *	1.59 (0.93–2.71)	1.68 (0.71–3.97)	2.08 (0.84–5.10)	1.30 (0.62–2.73)
No	1	1	1	1	1
**Sociodemographic characteristics**					
** Age (years)**					
18–30	1.38 (0.91–2.09)	2.27 (1.22–4.22) *	-	-	-
31–45	1.23 (0.81–1.86)	1.71 (0.94–3.11)	-	-	-
46–69	1	1	-	-	-
** Sex**					
Women	1.84 (1.28–2.66) **	1.21 (0.67–2.21)	0.98 (0.35–2.69)	1.40 (0.57–3.43)	0.66 (0.29–1.53)
Men	1	1	1	1	1
** Marital status**					
Never married	1.00 (0.68–1.45)	1.06 (0.61–1.83)	1.31 (0.65–2.64)	0.76 (0.31–1.86)	0.50 (0.17–1.43)
Separated/divorced	1.59 (0.95–2.65)	1.75 (0.87–3.51)	2.83 (1.01–7.92) *	2.58 (0.95–6.95)	0.48 (0.12–2.04)
Married	1	1	1	1	1
** Education level**					
Primary or lower	0.98 (0.65–1.47)	1.10 (0.61–1.97)	0.94 (0.37–2.41)	1.03 (0.33–3.22)	1.48 (0.35–6.18)
Secondary or lower	1.04 (0.71–1.51)	1.06 (0.62–1.80)	0.67 (0.28–1.59)	1.76 (0.72–4.32)	1.43 (0.37–5.43)
High school and higher	1	1	1	1	1
** Employment status**					
Currently unemployed	1.46 (1.07–1.99) *	1.67 (0.98–2.87)	1.59 (0.66–3.83)	2.13 (0.91–4.94)	1.51 (0.58–3.94)
Currently employed	1	1	1	1	1
** Place of residence**					
Urban	1.01 (0.74–1.37)	0.85 (0.51–1.41)	0.63 (0.24–1.65)	1.48 (0.73–3.00)	0.61 (0.20–1.84)
Rural	1	1	1	1	1
** Family history of suicide**					
Yes	1.99 (1.24–3.09) **	1.92 (1.13–3.67) *	2.02 (0.71–5.74)	2.17 (0.98–4.81)	2.23 (0.91–5.46)
No	1	1	1	1	1
**Abuse factors**					
** Childhood sexual abuse**					
Yes	4.03 (2.47–6.59)**	2.26 (1.10–4.66)*	3.59 (1.32–9.75)*	1.19 (0.22–6.40)	1.53 (0.48–4.89)
No	1	1	1	1	1
** Adult sexual abuse**					
Yes	3.80 (2.04–7.08)**	2.01 (0.84–4.79)	1.99 (0.54–7.32)	2.08 (0.56–7.73)	3.02 (0.96–9.40)
No	1	1	1	1	1
** Threats**					
Yes	2.36 (1.64–3.39)**	1.88 (1.15–3.06)*	1.69 (0.73–3.90)	1.83 (0.77–4.33)	1.90 (0.78–4.66)
No	1	1	1	1	1

cOR, crude odds ratio; aOR, adjusted odds ratio; CI, confidence interval

*p < 0.05; **p < 0.01; ***p < 0.005

^a^ Adjusted for all other listed variables in the model

^b^ Adjusted for all other listed variables in the model, excluding age

### Subgroup analysis on the association of chronic disease and lifestyle factors with past-year suicidal ideation among individuals with different sociodemographic characteristics

Our analysis revealed interactions between HBP and age (*p* < 0.03) and HBP and employment status (*p* < 0.002), which were significant enough to warrant a stratification analysis.

Further subgroup analysis (Tables 2 and 3) revealed that among individuals aged 18–30 years, those who were separated/divorced (aOR = 2.83, 95% CI = 1.01–7.92) and those who had experienced childhood sexual abuse (aOR = 3.59, 95% CI = 1.32–9.75) were more likely to have past-year suicidal ideation than their respective counterparts. Moreover, among individuals aged 31–45 years, HBP (aOR = 3.28, 95% CI = 1.36–7.88) was significantly associated with past-year suicidal ideation. Additionally, among currently employed individuals, HBP (aOR = 3.38, 95% CI = 1.54–7.40), no exercise (aOR = 2.65, 95% CI = 1.09–6.39), lifetime alcohol use (aOR = 2.40, 95% CI = 1.14–5.05), 18–30 years of age (aOR = 3.50, 95% CI = 1.01–12.1), and exposure to threats (aOR = 2.37, 95% CI = 1.01–5.53) were significantly associated with past-year suicidal ideation. Furthermore, among currently unemployed individuals, family history of suicide (aOR = 2.42, 95% CI = 1.07–5.45), childhood sexual abuse (aOR = 2.58, 95% CI = 1.24–5.38), and adult sexual abuse (aOR = 4.94, 95% CI = 1.94–12.5) were significantly associated with past-year suicidal ideation.

**Table 3:** Association of chronic diseases and lifestyle factors with suicidal ideation, stratified by employment status

Characteristics	Currently Employed aOR^a^ (95% CI)	Currently Unemployed aOR^a^ (95% CI)
**Chronic diseases**		
**High blood pressure**		
Yes	3.38 (1.54–7.40)**	0.87 (0.47–1.58)
No	1	1
**Cardiovascular disease**		
Yes	1.52 (0.57–4.06)	0.99 (0.41–2.41)
No	1	1
**Lifestyle factors**		
** Exercise**		
No	2.65 (1.09–6.39)*	1.15 (0.55–2.40)
Yes	1	1
** Currently smoking**		
Yes	1.57 (0.37–6.60)	1.58 (0.46–5.38)
No	1	1
** Lifetime alcohol use**		
Yes	2.40 (1.14–5.05)*	1.62 (0.86–3.08)
No	1	1
**Demographics**		
** Age (years)**		
18–30	3.50 (1.01–12.1)*	1.93 (0.10–0.86)
31–45	2.15 (0.72–6.42)	1.78 (0.72–6.42)
46–69	1	1
** Sex**		
Women	2.36 (0.93–5.99)	0.93 (0.39–5.99)
Men	1	1
** Marital status**		
Never married	0.97 (0.43–2.21)	1.54 (0.82–2.88)
Separated/divorced	1.40 (0.40–4.92)	2.21 (0.91–5.34)
Married	1	1
** Education level**		
Primary or lower	1.33 (0.49–3.63)	0.83 (0.39–1.78)
Secondary or lower	1.49 (0.71–3.13)	0.80 (0.39–1.63)
High school and higher	1	1
** Place of residence**		
Urban	0.81 (0.43–1.53)	0.89 (0.49–1.63)
Rural	1	1
** Family history of suicide**		
No	1.43 (0.65–3.11)	2.42 (1.07–5.45)*
Yes	1	1
**Abuse factors**		
** Childhood sexual abuse**		
Yes	2.37 (0.66–8.55)	2.58 (1.24–5.38)*
No	1	1
** Adult sexual abuse**		
Yes	0.53 (0.13–2.09)	4.94 (1.94–12.5)**
No	1	1
** Threats**		
Yes	2.37 (1.01–5.53)*	1.61 (0.77–3.38)
No	1	1

aOR, adjusted odds ratio; CI, confidence interval

*p < 0.05; **p < 0.01; ***p < 0.005

^a^Adjusted for all other listed variables in the model

## Discussion

Our findings contribute to the body of evidence for SSA and Eswatini regarding the association of chronic disease and lifestyle factors with past-year suicidal ideation. We examined the modifying role of sociodemographic characteristics in the association of chronic diseases and lifestyle factors with past-year suicidal ideation in Eswatini and revealed that the magnitude of past-year suicidal ideation was moderately high (9.9%) and somewhat comparable to the global average (9.2%) [7] and prevalence in Ethiopia (9%) [37], lower than that in South Africa (14.8%) [38], and higher than that in Nigeria (7.28%) [39]. The differences in suicidal ideation prevalence may possibly be explained by cultural influences and the varying survey instruments used across all studies.

We did not find any association of chronic diseases and lifestyle factors with past-year suicidal ideation, contrary to studies in developed [16, 40] and developing [20, 41] countries. Our findings differ from existing evidence for the SSA countries [40] but are consistent with the data for other LMICs [34]. The variation could be explained by cultural differences between countries.

We demonstrated the moderating role of age and current employment on the association of chronic disease and lifestyle factors with past-year suicidal ideation. First, HBP was associated with past-year suicidal ideation among adults aged 31–45, and second, HBP, lack of exercise, and lifetime alcohol use were associated with past-year suicidal ideation among currently employed individuals. Our findings underscore the importance of suicidal ideation intervention in people with chronic diseases and certain lifestyle behaviors, particularly among currently employed individuals, in the Eswatini population [11, 41].

Lack of exercise [42–44] and use of alcohol [21, 45] are established risk factors associated with suicidal ideation. Consistent with these findings, our study indicated that not exercising and lifetime alcohol use were associated with past-year suicidal ideation among the currently employed population. The relationship between alcohol use and suicidal ideation identified in this study accords with evidence from other developing [25, 46, 47] and developed [48, 49] countries. When people experience stressful life events that in turn result in suicidal ideation [50], they may use alcohol as a coping mechanism [51]. However, given our cross-sectional design, we limited our study to association rather than causal pathways. Furthermore, currently employed individuals, particularly those doing stressful jobs, are prone to drinking alcohol [52] and not exercising because they are stressed and busy due to work [53]. Nevertheless, we determined that currently employed individuals constitute a high-risk group for suicidal ideation, which is a novel finding in the context of SSA.

Our study demonstrated that HBP was associated with past-year suicidal ideation among currently employed individuals probably because of pressure at work [50, 54] or work dissatisfaction [55], which may impair an individual’s ability to cope, resulting in suicidal ideation. The findings are congruent with those in the literature [56]; however, the identification of currently employed individuals as a high-risk group for past-year suicidal ideation is unique.

Our findings are consistent with those of studies reporting that young age is associated with suicidal ideation [38, 57–59]. Most mental health problems, including suicidal ideation, occur among people in adolescence and are likely to persist through adulthood if not appropriately resolved [29, 60]. Moreover, the association of family history of suicide with past-year suicidal ideation was significant (*p* < 0.05) among individuals aged 31–45 years and currently unemployed individuals. The confluence of these factors can be explained in light of the premise that middle adulthood is the prime time of productivity in human development [61], and additional stressors increase the burden [62], which may influence suicidal ideation. In addition, current unemployment is considered a form of defeat, and a history of family suicide can exacerbate the burden, resulting in suicidal ideation [63]. Our study findings are consistent with those of studies conducted in other countries, which have reported that a family history of suicide is associated with suicidal ideation [8, 64, 65].

We demonstrated that, compared with being married, being separated or divorced was associated with past-year suicidal ideation, particularly among the young population. Our findings agree with those of a South Korea study; however, that study focused on comparing suicidal ideation between those with and without major depression [66]. Additionally, our data revealed a significant association of childhood sexual abuse among individuals aged 18–30 years and who were currently unemployed with past-year suicidal ideation. Such a relationship may develop because of the joint factors of accumulated negative childhood experiences [67] and being currently unemployed [68], which impair the ability of young adults to cope [69].

### Strengths and limitations

Our study used a large sample from a nationally representative survey that employed the WHO-recommended approach for assessing noncommunicable disease risk factors. The survey instruments were validated, and the country-level surveyors or data collection team members were well trained, yielding satisfactory quality control; moreover, the response rate was good. Such a robust survey methodology lends credibility to our findings. Moreover, because the present study employed a complex design to account for design, clustering, and sample weights, the results are generalizable to the Eswatini population. In general, we furnished novel findings (in the Eswatini context) on the association of HBP, physical exercise, and lifetime alcohol use with past-year suicidal ideation.

Our study entailed secondary analysis and a cross-sectional design in which the survey questions used to generate data were imprecise; therefore, our results must be interpreted with caution and be understood to reflect associations, not causation. However, survey questions have been used similarly in the literature to measure the same concepts by using single items [20, 70, 71]. Furthermore, to assess SES, it was estimated using household income. However, the data set contained many missing data points (67.2%) and was therefore omitted in the analysis; nevertheless, we incorporated education level [72] and employment status as proxies [73]. The measurement of lifestyle factors was not specific enough to yield good data; for example, the frequency and intensity of exercise were unclear, which may have led to exposure underestimation. Moreover, suicidal ideation related questions were not specific enough to properly classify those with and without suicidal ideation because responses were subject to various interpretations by the respondents. Data on all variables, including suicidal thoughts, HBP, and smoking history, were self-reported, which may be subject to recall bias. Furthermore, respondents may furnish socially acceptable but inaccurate answers, particularly for questions on chronic diseases, lifestyle factors, and suicidal thoughts, for fear of being judged.

Our study has some critical implications that can guide strategic policy decisions. Eswatini lacks a suicide prevention policy and has no priority indicators of suicidal behaviors in client management information systems (CMISs) [74]. Therefore, the development and integration of such indicators into CMISs is necessary. In brief, a CMIS is a surveillance database system that is under the custody of the health ministry in Eswatini, and data are deidentified and stored in servers. Although debates are ongoing regarding surveillance and records of mental health and health behaviors, they are in line with the WHO mental health action plan [9, 75]. Although the integration of suicidal behavior and other lifestyle indicators is advocated, it raises ethical concerns, which must be considered [76].

Physical exercise should be promoted among currently employed individuals to improve their health. Consistent with previous studies, our study demonstrated that lifetime alcohol use is associated with past-year suicidal ideation among the currently employed population [47]. Our findings can form the basis for the design and implementation of suicidal ideation prevention or intervention programs, particularly for the high-risk groups identified. Furthermore, the implementation and integration of suicidal behavior indicators to monitoring and evaluation systems could enhance the identification and characterization of high-risk groups (e.g., those aged 31–45 years and those who are unemployed, as identified in our study) for suicidal behavior [77].

## Conclusions and recommendations

Our findings indicated that HBP, lack of exercise, and lifetime alcohol use among currently employed individuals were associated with past-year suicidal ideation. Our study results form the basis for a call to action for better survey research in Eswatini (by using more validated scales than a single item); moreover, adopting longitudinal cohort designs will allow the establishment of causality in suicidal behavior studies. Furthermore, we advocate for mental health surveys to understand the mechanisms underlying this association among different employment codes. Studies should consider more comorbid conditions, such as other common mental health issues (e.g., depression) and chronic diseases (e.g., human immunodeficiency virus), and include general health information.

## Data Availability

The datasets used or analyzed were with permission from the WHO NCD Microdata Repository, which serves as custodian of publicly available data; data are available on the website https://extranet.who.int/ncdsmicrodata/index.php/catalog.
